# N6-methyladenosine with immune infiltration and PD-L1 in hepatocellular carcinoma: novel perspective to personalized diagnosis and treatment

**DOI:** 10.3389/fendo.2023.1153802

**Published:** 2023-07-03

**Authors:** Yanlong Shi, Yizhu Wang, Wenning Zhang, Kaiyi Niu, Xinyu Mao, Kun Feng, Yewei Zhang

**Affiliations:** Hepatopancreatobiliary Center, The Second Affiliated Hospital of Nanjing Medical University, Nanjing, Jiangsu, China

**Keywords:** N6-methyladenosine, hnRNPC, PD-L1, hepatocellular carcinoma, prognosis, immune infiltration, immunotherapy

## Abstract

**Background:**

Increasing evidence elucidated N6-methyladenosine (m6A) dysregulation participated in regulating RNA maturation, stability, and translation. This study aimed to demystify the crosstalk between m6A regulators and the immune microenvironment, providing a potential therapeutic target for patients with hepatocellular carcinoma (HCC).

**Methods:**

Totals of 371 HCC and 50 normal patients were included in this study. GSE121248 and GSE40367 datasets were used to validate the expression of HNRNPC. The R package “ConsensusClusterPlus” was performed to screen consensus clustering types based on the expression of m6A regulators in HCC. The R package “pheatmap”, “immunedeconv”, “survival”, “survminer” and “RMS” were applied to investigate the expression, immunity, overall survival, and clinical application in different clusters and expression groups. Comprehensive analysis of HNRNPC in pan-cancer was conducted by TIMER2 database. Besides, HNRNPC mRNA and protein expression were verified by qRT-PCR and immunohistochemistry analysis.

**Results:**

Most of m6A regulators were over-expressed excerpt for ZC3H13 in HCC. Three independent clusters were screened based on m6A regulators expression, and the cluster 2 had a favorable prognosis in HCC. Then, the cluster 2 was positively expression in macrophage, hematopoietic stem cell, endothelial cell, and stroma score, while negatively in T cell CD4^+^ memory and mast cell. We identified HNRNPC was an independent prognostic factor in HCC, and nomogram performed superior application value for clinical decision making. Moreover, PD-L1 was significantly up-regulated in HCC tissues, cluster 1, and cluster 3, and we found PD-L1 expression was positively correlated with HNRNPC. Patients with HCC in high-expression groups was associated with tumor-promoting cells. Besides, HNRNPC was correlated with prognosis, TMB, and immune checkpoints in cancers. Particularly, the experiments confirmed that HNRNPC was positively expression in HCC cells and tissues.

**Conclusion:**

The m6A regulators play irreplaceable roles in prognosis and immune infiltration in HCC, and the relationship of HNRNPC and PD-L1 possesses a promising direction for therapeutic targets of immunotherapy response. Exploration of m6A regulators pattern could be build the prognostic stratification of individual patients and move toward to personalized treatment.

## Introduction

Liver cancer is the sixth most common cancer in humans and the fourth leading cause of cancer-related death worldwide (1). Hepatocellular carcinoma (HCC) is characterized by rapid progression and poor prognosis, accounting for 90% of primary liver cancer (2). HCC can be attributed to adverse factors, mainly including hepatitis B virus, alcohol, and aflatoxin (3). Moreover, most HCC patients suffer from recurrence and metastasis due to tumor heterogeneity, resulting in a poor prognosis (4). Immunotherapy, a promising therapeutic strategy, refers to artificially enhancing or inhibiting body’s immune function to treat diseases (5). Although immunotherapy has fewer toxic side effects than chemotherapy, the blocking of the immune checkpoints by immune microenvironment is still the leading cause of poor prognosis in HCC patients (6). Furthermore, the expression of PD-L1 is regulated by adverse of factors, resulting in different meanings of PD-L1 positivity1. Hence, it is essential to further understand the molecular mechanisms of immunotherapy of HCC and improve the therapeutic effect.

N6-methyladenosine (m6A) modification, the most abundant internal modification of RNA in eukaryotic cells, is attracting wide attention from researchers (7). m6A methylation is regulated by regulatory factors: writer, reader and eraser, which contribute to physiological and pathological occurrence (8). It affects almost every aspect of RNA metabolism, playing a crucial role in regulating RNA maturation, stability, and translation (9). It was reported that IGF2BP1, a crucial m6A-dependent manner, might be a novel drug candidate for cancer therapeutics by modulating tumor immune microenvironment in m6A regulation (10). Increasing evidence reported that m6A dysregulation participated in various cancers, including HCC (11, 12). For example, methyltransferase-like 3 (*METTL3)*, the critical component of m6A RNA methyltransferase, was obviously up-regulated in HCC, and knockdown of *METTL3* could weaken lung metastasis (11). Moreover, an important study elaborated on the vital role of m6A in both primitive and adaptive immune responses, suggesting the potential role of m6A in tumor immunity (13). The combination of m6A regulators and programmed cell death protein 1 (*PD-1)* inhibitors was required to maintain of cell and tissue homeostasis, and had a synergistic effect to enhance the efficacy of cancer immunotherapy (14). Although progress has been made in the modification of m6A in HCC, its mechanism in tumor immunotherapy remains unclear. Therefore, demystifying the crosstalk between m6A regulators and the immune microenvironment could be a potential therapeutic target for HCC patients.

In this study, we comprehensively assessed the roles of m6A regulators based on molecular subtypes in HCC, and identified and validated a key m6A regulator *HNRNPC* in different datasets and basic experiments. Moreover, we systematically undertook the relationship of *HNRNPC* and *PD-L1* in expression, prognosis, and immune microenvironment.

## Materials and methods

### Data acquisition

The RNA-seq data and relevant information were obtained from The Cancer Genome Atlas (TCGA) database (https://portal.gdc.cancer.gov/), with 371 HCC and 50 normal tissues. Pan-cancer analysis included totals of 33 types tumors and adjacent normal tissues in TIMER2 database. GSE121248 and GSE40367 datasets were used to validate the expression of HNRNPC (https://www.ncbi.nlm.nih.gov/geo/) (15, 16).

### Analysis and evaluation of m6A regulators consensus clustering

The R package “ConsensusClusterPlus” was used for consensus analysis for HCC, and the parameter was set as clusterAlg = “hc”, innerLinkage=‘ward.D2’ (17). The cluster heatmap was analyzed by R package “pheatmap”. The gene expression heatmap retained genes with variance above 0.1. In correlation analysis, the circles represent genes related to m6A, the lines represent the interrelationships between genes, and the different colors of the circles represent different clustering categories. Among them, red and blue represent positive and negative correlation respectively. The prognosis of various clusters was determined by Kaplan-Meier survival curve in HCC, following HR with 95% confidence interval. The R package “immunedeconv” was applied to immune infiltration cells and score between cluster 1 and cluster 2 (18). Then, we extracted the expression values of immune checkpoint-related genes from RNA-seq data to observe the expression of immune checkpoint-related genes in different clusters.

### Identification, validation, development of key m6A regulators

The key m6A regulator was identified by Venn diagram. The boxplot of gene expression was plotted using R package “Boxplot”. The R package “survival” and “survminer” was performed to compare the survival differences between the two or more groups, and the timeROC analysis was used to compare the prediction accuracy. The Kaplan-Meier plot database was conducted to investigate overall survival (OS), progression free survival (PFS), and disease free survival (DFS) in HCC. The univariate and multivariate cox regression estimated the independent prognostic value, then presented it by R package “forestplot”. Based on the results of multivariate Cox regression, the R package “RMS” developed the nomogram to predict survival probability. The proportion of immune infiltration cells was calculated by CIBERSORT algorithm.

### Cell culture

The hepatic normal cell (LO2) and HCC cell line (HepG2) were donated from School of Basic Medicine, Anhui Medical University. All cells were cultured with DMEM containing 10% fetal bovine serum (VivaCell, Shanghai, China), and culture conditions were as follows: 5% CO_2_, 37°C.

### Quantitative real-time polymerase chain reaction

Experimental procedures were performed as in previous studies (19). The SYBR Green qPCR Mix (Takara) was used to quantitative *HNRNPC* expression in different groups compared to *GADPH* expression. The results were calculated as 2^−ΔΔCt^ method. All primer sequences were as follows: *HNRNPC*:5’-aattgtgggctgctctgttc-3’; 3’-aacctggccagcaatcattc-5’, *GADPH*: 5’-CTCACCGGATGCACCAATGTT-3’; 3’-CGCGTTGCTCACAATGTTCAT-5’.

### Analysis of immunohistochemistry

The detailed procedure of Immunohistochemistry in a previous study has been described (20). *HNRNPC* protein expression was detected by immunohistochemistry in normal and HCC tissues. In the HPA database, we detected the image of *HNRNPC* expression in “tissue” and “pathology” modules (21). All results were re-judged by two pathologists. Regents as follows: *HNRNPC*: Atlas Antibodies Cat#AMAb91010, RRID : AB_2665761, dilution 1:1500.

### Statistical analysis

The version 3.8.1 was used for all R packages. The Spearman analysis was applied to the correlation among m6A regulators. The significance of two groups was determined by the Wilcox test, and the significance of three groups or more was determined by the Kruskal-Wallis test. The log rank test was used for survival differences. The expression of *HNRNPC* was analyzed by Student’s t-test in tissues and cell lines. *P*<0.05 was defined as a statistical difference.

## Results

### Differences in the expression of m6A regulators between HCC and normal tissues

**Figure 1:**
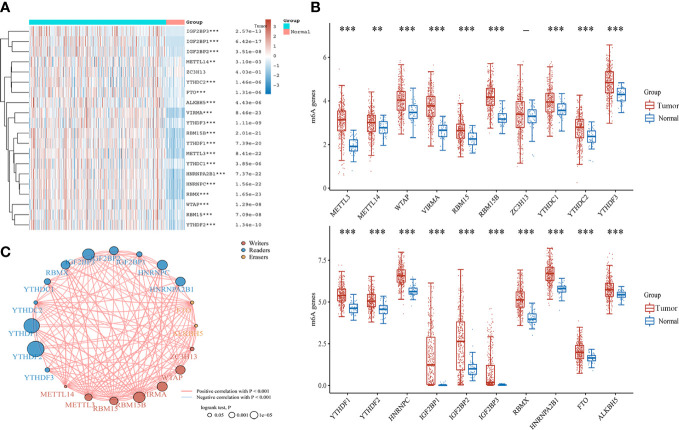
The expression and correlation of m6A regulators in HCC. **(A)** Heatmap of m6A regulators between HCC and normal tissues. **(B)** The expression of m6A regulators in HCC and normal tissues by Box plot analysis. **(C)** The correlation of m6A regulators in HCC by Spearman analysis. **P<0.01, ***P<0.001.

Totals of 20 m6A regulators across 33 cancer types were obtained from previous study (17) (**Supplement Table 1**). To understand the role of m6A regulators in HCC, we investigated the expression of m6A regulators between 371 HCC and 50 normal tissues in the TCGA database. It was found that the majority of m6A regulators was up-regulated in HCC except *ZC3H13*, including *METTL3*, *METTL4*, *WTAP*, *VIRMA*, *RMB15B*, *YTHDC1*, *YTHDC2*, *YTHDF3*, *YTHDF1*, *YTDHF2*, *HNRNPC*, *IGF2BP1*, *IGF2BP2*, *IGF2BP3*, *RBMX*, *HNRNPA2B1*, *FTO*, and *ALKBHS* (**Figures 1A, B**). A correlation analysis indicated a positive correlation among writers, readers, and erasers of m6A (**Figure 1C**). These findings suggest that m6A regulators might act as a vital role in mediating the development and progression of HCC.

### Identification and evaluation of HCC subtypes based on m6A regulators

**Figure 2:**
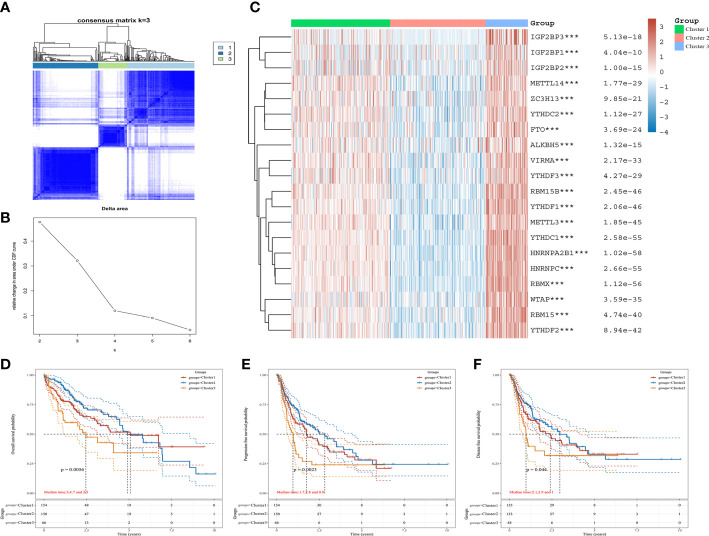
The expression and prognosis of m6A regulators of HCC in different clusters. **(A)** The optimal consensus clustering matrix k=3. **(B)** The relative change in area under cumulative distribution function curves. **(C)** The expression of m6A regulators in three clusters of HCC by heatmap. **(D–F)** The prognostic value for three clusters of HCC patients by Kaplan–Meier curves. **(D)** Overall survival. **(E)** Disease-free survival. **(F)** Progression free survival. ***P<0.001.

Based on the expression of m6A regulators, we applied to consensus clustering analysis in HCC. In **Figure 2A**, the optimal matrix value shows a good distribution in different parts. From the relative change in area under cumulative distribution function curves, the delta area of 3~4 was steepest from k=2 to 6 (**Figure 2B**). Therefore, the k value of 3 was conducted to follow-up analysis. The baseline characteristics of the three clusters and associations are presented in **Table 1**. Then, the expression difference in three clusters was shown by heatmap analysis. We found that the cluster 1 and cluster 3 were up-regulated in m6A regulators of HCC, but down-regulated in cluster 2 (**Figure 2C**). Moreover, Kaplan–Meier curves suggested that patients with cluster 2 had favorable overall survival (*P* < 0.05) (**Figure 2D**), progression-free survival (*P* < 0.05) (**Figure 2E**), and disease free survival (*P* < 0.05) (**Figure 2F**) than cluster 1 and cluster 3. These results revealed an obvious distinction among cluster 1, cluster 2, and cluster 3 of patients with HCC.

**Table 1:** Clinical characteristics of three clusters of patients with HCC.

Characteristics		Cluster 1 (n=155)	Cluster 2 (n=150)	Cluster 3 (n=66)	*P*_value
Status	Alive	101 (65.2%)	103	37	0.201
	Dead	54 (34.8%)	47	29	
Age	Mean (SD)	61.3 (12.6)	59.8 (13.3)	54.2 (14.9)	0.004
Gender	FEMALE	52	44	25	0.443
	MALE	103	106	41	
Race	AMERICAN INDIAN	1	1		0.266
	ASIAN	66	60	32	
	BLACK	11	3	3	
	WHITE	75	78	31	
	Not known	2	8		
Grade	G1	23	28	4	0.006
	G2	73	80	24	
	G3	51	38	33	
	G4	4	4	4	
	Not known	4		1	
T stage	T1	80	82	19	0.056
	T2	37	36	21	
	T3	16	13	16	
	T3a	12	11	6	
	T3b	3	2	1	
	T4	6	5	3	
	Not known	1	1		
N stage	N0	108	97	47	0.288
	N1	1	1	2	
	NX	46	52	16	
	Not known			1	
M stage	M0	111	108	47	0.936
	MX	44	39	18	
	M1		3	1	
pTNM_stage	I	73	79	19	0.028
	II	34	35	17	
	IIIA	27	20	18	
	IIIB	5	2	1	
	IIIC	3	2	4	
	III		1	2	
	IV		4	1	
	Not known	13	7	4	

### Relationship of m6A subtypes with immune microenvironment and PD-L1 expression in HCC

**Figure 3:**
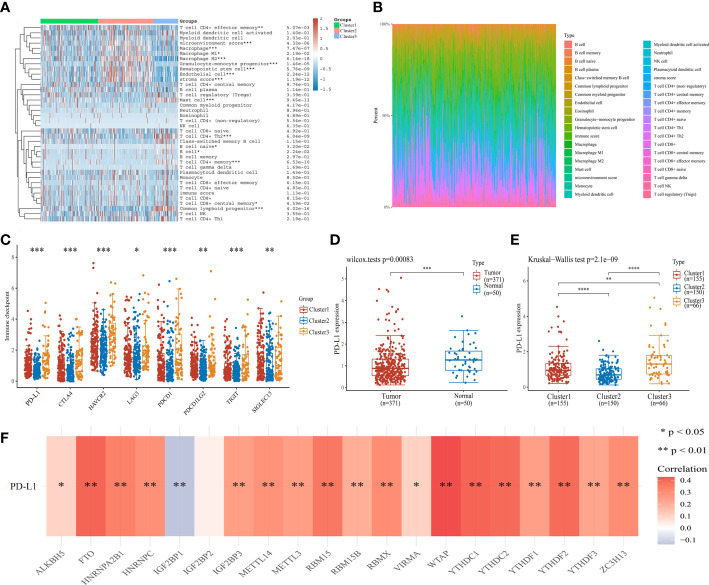
Association of cluster subtypes with immune microenvironment and *PD-L1* expression in HCC. **(A)** The relationship between m6A subtypes and immune cell infiltration level in HCC by heatmap analysis. **(B)** Proportion of immune infiltration cells in different cluster groups. **(C)** The relationship between cluster subtypes and immune checkpoint expression in HCC. **(D)** *PD-L1* expression in HCC and normal tissues. **(E)** The difference of *PD-L1* expression in cluster 1, cluster 2, and cluster 3. **(F)** The correlation of m6A regulators and *PD-L1* expression. *P<0.05, **P<0.01, ***P<0.001, and ****P<0.0001.

To determine the association between cluster subtypes and immune microenvironment, we first explored the difference in immune cell infiltration levels in three clusters using by XCELL algorithm. The cluster 2 was positively expressed in macrophage, hematopoietic stem cell, endothelial cell, microenvironment score, and stroma score, while negative in T cell CD4+ memory and mast cell (**Figure 3A**). The three clusters were lower expression in B cell naive. The corresponding proportion of immune infiltration cells is presented in **Figure 3B**. Then, the connection of immune checkpoints and cluster subtypes was assessed by heatmap of gene expression in HCC, and the results showed a significant difference in the expression of immune checkpoints and cluster subtypes (**Figure 3C**). Then, we selected *PD-L1* for further study. Compared with normal tissues and cluster 2, the expression of *PD-L1* was significantly up-regulated in HCC tissues and the other two clusters (**Figures 3D, E**). Moreover,we further analyzed the correlation between m6A regulators and *PD-L1* expression. *PD-L1* expression was positively correlated with m6A regulators, mainly including *FTO*, *HNRNPA2B1*, *HNRNPC*, *RBM15*, *WTAP*, *YTHDC1*, and *YTHDF2*, while negatively in *IGF2BP1* (**Figure 3F**).

### The m6A regulator *HNRNPC* was over-expressed in HCC

**Figure 4:**
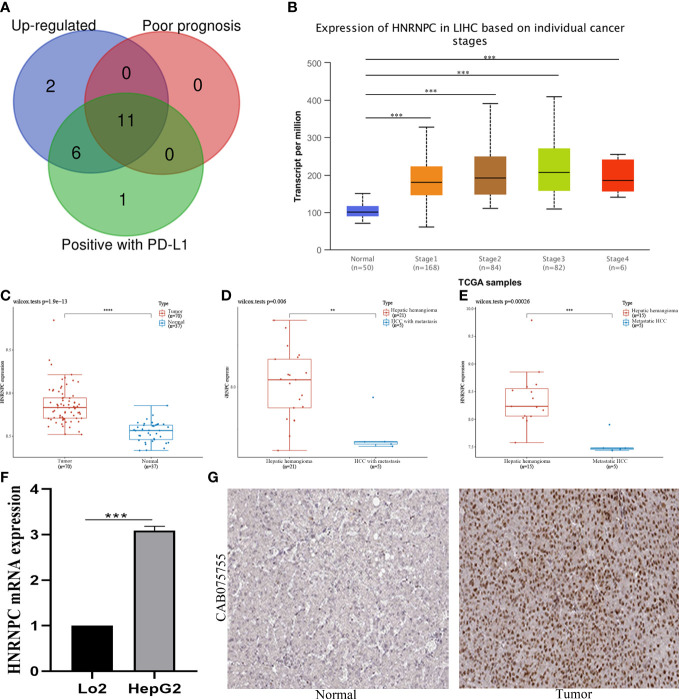
Identification and validation of *HNRNPC* expression in HCC. **(A)** Screening of 11 hub genes among up-regulated genes, genes of poor prognosis, and genes with *PD-L1* positively by venn graph. **(B)** Expression of *HNRNPC* in HCC based on individual cancer stages. **(C)** *HNRNPC* expression in 70 HCC tissues and 37 normal tissues in GSE121248 database. **(D)** *HNRNPC* expression in 21 hepatic hemangioma tissues and 5 HCC with metastatic tissues in GSE40367 database. **(E)** *HNRNPC* expression in 15 hepatic hemangioma tissues and 5 metastatic HCC in GSE40367 database. **(F)** *HNRNPC* expression in HCC cell lines by qRT-PCR analysis. **(G)** *HNRNPC* protein expression in HCC and normal tissues by HPA database. **P<0.01, ***P<0.001, and ****P<0.0001.

To further investigate the potential effects of m6A regulators in HCC, we conducted an intersection analysis to screen key m6A regulators among up-regulated expression of HCC, positively correlated with the expression of *PD-L1*, and associated with worse prognosis. We identified 11 relevant genes (**Figure 4A**). However, only *HNRNPC* expression was correlated with tumor stages (**Figure 4B**). These results indicated that *HNRNPC* might play a vital role in HCC progression and metastasis. In **Figure 4C**, we first validated the expression of *HNRNPC* between HCC and normal tissues in GSE121248 database. The result showed *HNRNPC* expression was over-expressed in HCC. Subsequently, compared with hepatic hemangioma tissues, *HNRNPC* expression was also significantly increased in HCC with metastasis tissues, and metastatic HCC tissues respectively based on GSE40367 database (**Figures 4D, E**). Moreover, our experiment demonstrated the expression of *HNRNPC* was up-regulated in HCC cell lines by qRT-PCR analysis (**Figure 4F**). Notably, the protein expression of *HNRNPC* was detected by HPA database. *HNRNPC* protein was positively expressed in HCC tissues compared to normal tissues, and was mainly located in nuclear (**Figure 4G**).

### *HNRNPC* independent prognostic value in HCC

**Figure 5:**
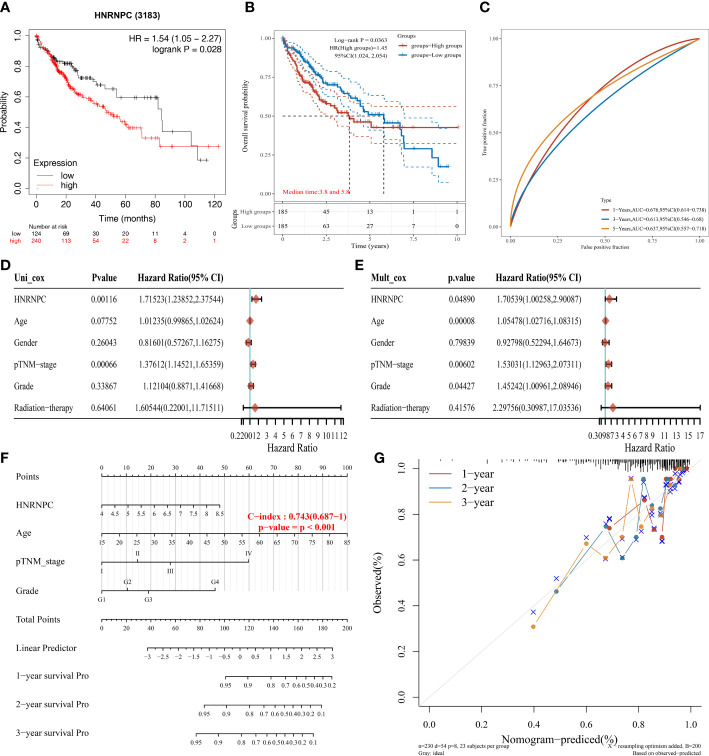
Independent prognostic value of *HNRNPC* in *HCC*. **(A)** The prognosis of HCC patients with low and high *HNRNPC* expression by Kaplan-Meier plotter database. **(B)** The overall survival probability of *HNRNPC* in HCC patients of TCGA database. **(C)** The AUC value of *HNRNPC* in 1-, 3-, and 5-years. **(D)** The univariate Cox regression between *HNRNPC* expression and clinicopathological characteristics. **(E)** The multivariate Cox regression between *HNRNPC* expression and clinicopathological characteristics. **(F)** Evaluation of overall survival in 1-, 2-, and 3-years for HCC patients by nomogram. **(G)** Calibration curve of the nomogram model.

The Kaplan-Meier plotter showed the low expression group had a better prognosis (**Figure 5A**). In **Figure 5B**, HCC patients in low expression group had a longer survival than high expression group. Then we further explored the relationship between *HNRNPC* expression and overall survival. Subsequently, the AUC values of *HNRNPC* expression in 1-, 3-, and 5-year were 0.676, 0.613, and 0.637 for forecasting survival (**Figure 5C**). Moreover, forest plots of univariate and multivariate Cox analysis suggested *HNRNPC* expression (*P*=0.001, HR=1.715; P=0.049, HR=1.705), and pTNM−stage (*P<*0.001, HR=1.376; P=0.006, HR=1.530) was associated with OS (**Figures 5D, E**). Besides, to evaluate the predictive efficiency of *HNRNPC* expression, we established the nomogram to predict 1-, 2-, and 3-years survival probability, with the value of C-index as 0.743(0.687−1) (**Figure 5F**). The calibration curve of nomogram performed a superior application for clinical decision making (**Figure 5G**). These results revealed that *HNRNPC* could serve as a novel independent prognostic biomarker for HCC patients.

### Analysis of the correlation between *HNRNPC* expression and PD-L1, and immune infiltrating cells

**Figure 6:**
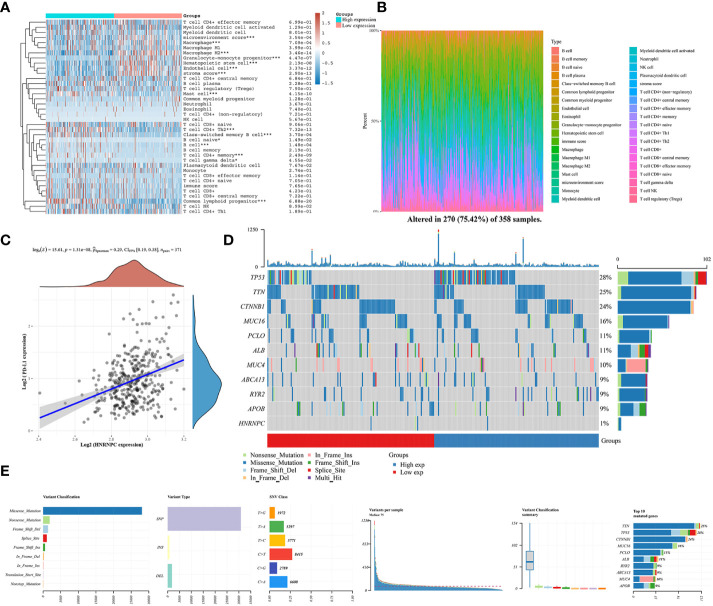
The relationship between immune microenvironment and HNRNPC expression groups and HNRNPC somatic landscape in HCC. **(A)** Analysis of immune cell infiltration level and low and high HNRNPC expression in HCC by heatmap analysis. **(B)** Proportion of immune infiltration cells in low and high HNRNPC expression groups. **(C)** The correlation of HNRNPC expression and PD-L1. **(D)** Lollipop charts of the mutated HNRNPC gene. **(E)** The variation distribution of variant classifications, types and SNV class in HCC. *P<0.05, ***P<0.001.

We explored the relationship between immune infiltration cells and *HNRNPC* low- and high-expression groups by CIBERSORT algorithm. Patients in high-expression groups were increased in mast cell, T cell CD4+ Th2, class-switched memory B cell, B cell naive, T cell CD4+ memory, and common lymphoid progenitor, but decreased in microenvironment score, macrophage M2, granulocyte-monocyte progenitor, hematopoietic stem cell, and stroma score (**Figure 6A**). Moreover, the proportional abundance of immune infiltrating cells was visualized by heatmap in *HNRNPC* low- and high-expression groups (**Figure 6B**). To definite the role of immunity in *HNRNPC* expression, we conducted a correlation analysis between *HNRNPC* expression and *PD-L1* expression in HCC. *HNRNPC* was positively correlated with *PD-L1* expression in HCC (*P*=1.31e-08, Spearman=0.29) (**Figure 6C**). The Oncoplot showed the somatic landscape of HCC, altered in 270 (75.42%) of 358 samples (**Figure 6D**). *TP53* mutation is most common, followed by *TTN* and *CTNNB1* mutations. The mutation pattern of *HNRNPC* was Nonsense_Mutation and Missense_Mutation, accounting for 1%. In **Figure 6E**, We further exhibit the variation distribution of variant classifications, types and SNV class, so as to provide novel theories into immunotherapy for different risk groups.

### Comprehensive analysis of *HNRNPC* in pan-cancer

**Figure 7:**
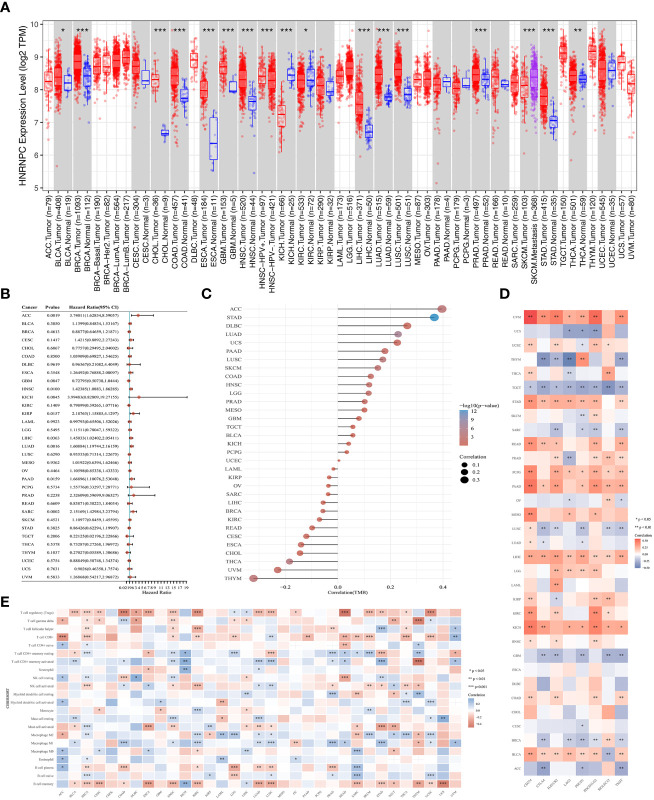
Comprehensive analysis of key m6A regulator HNRNPC in pan-cancer. **(A)** The expression of HNRNPC was up-regulated in various cancers. **(B)** The prognosis of HNRNPC in pan-cancer by forest plot. **(C)** The association between tumor mutation load and HNRNPC expression. **(D)** The correlation of HNRNPC expression with immune checkpoints in pan-cancer. **(E)** The correlation of HNRNPC expression with immune infiltrating cells in pan-cancer. *P<0.05, **P<0.01, and ***P<0.001.

To reveal the potential role of m6A regulator *HNRNPC*, we further analyzed *HNRNPC* expression, prognosis, TMB, immune checkpoints, and immune infiltration cells in pan-cancer. The up-regulation in *HNRNPC* expression was shown among 17 types cancers (**Figure 7A**). Importantly, we noticed that *HNRNPC* was down-regulated in Kidney chromophobe compared with normal tissues. Then, the forest suggested that *HNRNPC* had a poor prognosis in Adrenocortical Carcinoma (ACC), Head and Neck Squamous Cell Carcinoma (HNSC), Kidney Renal Papillary Cell Carcinoma (KIRP), LIHC, Lung Adenocarcinoma (LUAD), Pancreatic Adenocarcinoma (PAAD), and Sarcoma (**Figure 7B**). TMB has been considered as a quantifiable and profound immune response biomarker to forecast immunotherapy effects (22). The expression level of *HNRNPC* was associated with TMB in several cancers, including STAD, LUAD, Lung Squamous Cell Carcinoma (LUSC), Skin Cutaneous Melanoma (SKCM), Thyroid Carcinoma (THCA), Thymoma (THYM) (**Figure 7C**). Moreover, we estimated the relationship between the *HNRNPC* expression and immune checkpoints, and the results identified that the expression of HNRNPC was positively associated with most immune checkpoints in BLCA, LIHC, PAAD, PCPG, STAD, UVM, while negatively correlated with immune modulators in BRCA, GBM, LUSC, TGCT and THYM (**Figure 7D**). In addition, we observed T cell regulatory (Tregs), CD8+ T cell, NK cell activated, CD4+ T cell, Macrophage M1, and B cell memory were linked with the *HNRNPC* expression (**Figure 7E**).

## Discussion

The emergence of immunotherapy, aiming to eradicate malignant cells and reinforce human immune system, is a revolutionary innovation for cancer treatment (23). Recently, with RNA modification coming into scientific arena, m6A modification, as a critical process in transcript expression, has collected enormous interest. Amounts studies have illustrated that the aberrant expression of m6A regulators is involved in cancer formation and progression, thus providing a new direction for immunotherapy (24). For example, m6A modification affects the *IL-7/STAT* pathway by regulating the mRNA of *SOCS* family genes to influence T cells (25). Moreover, the downregulation of *METTL14* was involved in tumor metastasis, and it performed an adverse prognostic factor for survival without recurrence in HCC patients (26). However, the relationship of m6A regulator and *PD-L1* is not fully elaborated.

Herein, we identified different m6A regulators subtypes by consensus clustering based on the differential expression of 20 m6A regulators in HCC. Then, we performed the relationship between three independent clusters and different clinicopathological characteristics, prognosis, immune microenvironment, and *PD-L1* in HCC. The cluster 2 was down-regulated expression in HCC, suggesting that patients with cluster 2 had a favorable prognosis by Kaplan-Meier curves analysis. An adequate characterization and validation determined *HNRNPC* as a prognosis biomarker and immune infiltration-related m6A regulator in HCC by public databases, qRT-PCR and immunohistochemistry analysis. Moreover, the pan-cancer analysis further demonstrated the comprehensive landscapes of *HNRNPC* in different cancers.

*HNRNPC*, a key m6A regulator belonging to the hnRNP family, regulates multiple functions in RNA splicing, RNA expression, RNA stabilization, and RNA translation (27–29). Increasing evidence suggested that up-regulation *HNRNPC* expression was associated with the occurrence and progression of tumors, such as breast cancer (30), gastric cancer (31), and glioblastoma (32). *HNRNPC* expression was correlated with tumor stage, lymph node metastasis, and poor prognosis in oral squamous cell carcinoma (33). Moreover, HNRNPC impairs vascular endothelial function and promotes the occurrence of vascular complications in type 2 diabetes (34). In our study, we found that *HNRNPC* was up-regulated in HCC tissues compared to normal tissues, and this result was validated by GSE121248 and GSE40367 datasets. Importantly, the qRT-PCR and immunohistochemistry analysis suggested the expression level of *HNRNPC* was over-expressed in HCC cells, and *HNRNPC* protein was positively expressed in HCC tissues. The Kaplan-Meier plotter showed the low expression group had a better prognosis. Furthermore, the univariate and multivariate Cox analysis indicated *HNRNPC* expression was an independent prognostic factor. Besides, based on *HNRNPC* expression and clinical features, we established the nomogram to evaluate the predictive efficiency, and the results performed a superior application for clinical decision making. However, the underlying function and mechanism need to exploit in HCC in detail.

The combination of m6A regulators and immune inhibitors have an active influenced on cancer immunotherapy efficacy. In this study, patients with HCC were classified into three clusters based on m6A regulators. Various differences in immune infiltration cells among three clusters with HCC were identified by the XCELL algorithm. For example, the infiltration levels with anti-tumor cells like T cell CD4+ effector memory, endothelial cell, and macrophage M2 were significantly up-regulated in cluster 2. Meanwhile, tumor-promoting cells like mast cell, T cell CD4+ Th2, T cell CD4+ memory, and T cell CD8+ central memory were higher in HCC patients with cluster 1 and cluster 3. The expression of Treg cells and NK cells was no difference in different clusters and expression groups. However, Li et al. reported that HNRNPC regulated the activation of Treg cells by activating the immune microenvironment, which may be a potential therapeutic target for prostate cancer (35). In pancreatic cancer, HNRNPC induced DNA damage repair and cancer-associated fibroblast activation through the RhoA/ROCK2-YAP/TAZ signaling pathway (36). Moreover, we also investigated the relationship between *HNRNPC* expression and immune infiltration cells based on *HNRNPC* low- and high-expression groups. Interesting, the results of infiltration levels in patients with low *HNRNPC* expression were consistent with cluster 2. These findings are in accordance with the role T cell CD4+ effector memory (37) and endothelial cell (38) act in regulating anti-tumor responses. Furthermore, previous studies reported mast cell promoted tumor growth and invasion in the tumor microenvironment, leading to poor overall clinical prognosis (39). Our results confirmed that m6A regulators in low-expression group could enhance tumor immune microenvironment to kill tumors. Notably, this study is the first to reveal the correlation between *HNRNPC* and *PD-L1* in HCC, resulting be a potential biomarker for prognosis, and offering novel theory for immunotherapy response and therapeutic target related to *PD-L1*.

TMB could be consider a potential immunotherapy parameter that can determine patients responsiveness to immune checkpoint blockers (40). The higher mutation rate in patients with HCC provides novel insights into immunotherapy in different risk groups. Moreover, the immune microenvironment of HCC mainly involved the upregulation of *PD-L1* and *PD-L2* in Kupffer cells, hepatic sinus endothelium and leukocyte (41). It was reported that inflammatory responses with overexpression of *PD-1* and *PD-L1* were detected in 25% of HCC patients (6). Tumor cells can use *PD-L1* to bind to the *PD-1* of T cells, evading recognition and allowing them to continue to spread throughout the body (42). We found that *PD-L1* expression was significantly up-regulated in HCC tissues and other two clusters, and there was a closely relationship between *PD-L1* and m6A regulators. Moreover, *HNRNPC* expression was positively correlated with *PD-L1* expression in HCC. The combination of anti-*PD-L1* and other therapy strategies is gradually improving the prognosis of advanced cancers in HCC, maintaining its ability to recognize and kill tumor cells (43). Besides, comprehensive analysis indicated that the key m6A regulator, *HNRNPC*, is not only a novel prognostic biomarker in multiple cancers, but also regulates tumor immune microenvironment and immune checkpoints, providing a vital opportunity for developing immune targets.

This study has some potential limitations that deserves to be noticed. Firstly, m6A regulators were obtained from previous study, some m6A regulators may have not been included in this study. Secondly, although the TCGA cohort was used to clustering and grouping, further validation is still needed in multi-center and prospective cohorts in the future. Besides, it is necessary to explore the biological function and mechanism between m6A key regulator *HNRNPC* and *PD-L1* in HCC *in vivo* and vitro.

## Conclusion

An extraordinary analysis was undertaken, referring to the expression and the relationship with prognosis, immune microenvironment, and *PD-L1* of m6A regulators in HCC based on different clusters and expression groups. The key m6A regulator *HNRNPC* could be a prognostic biomarker, which discloses the association between *HNRNPC* and immune microenvironment in HCC. Further exploration should focus on the potential mechanisms by which *HNRNPC* modulates the immunotherapy and offer a novel theory for therapeutic targets related to PD-L1 in HCC.

## Data availability statement

The datasets presented in this study can be found in online repositories. The names of the repository/repositories and accession number(s) can be found in the article/**Supplementary Material**.

## Author contributions

YS, YW, and WZ conceived and designed the study, revised the manuscript. YS,YW, and KN wrote the manuscript. WZ and XM extracted and analyzed the data alone. KF and KN performed the experiments. YZ edited the manuscript and funded this study. All the authors read and approved the final manuscript.
